# Adjuvant Efficacy of the ECMS-Oil on Immune Responses against *Bordetella bronchiseptica* in Mice through the TLR2/MyD88/NF-*κ*B Pathway

**DOI:** 10.1155/2023/1011659

**Published:** 2023-03-27

**Authors:** Chenwen Xiao, Yee Huang, Xuemei Cui, Qiang Wei, Quanan Ji, Yan Liu, Su Fei, Yao Pan, Xiangfei Xu, Huang Pan, Guolian Bao

**Affiliations:** Institute of Animal Husbandry and Veterinary Science, Zhejiang Academy of Agricultural Sciences, Hangzhou, Zhejiang, China

## Abstract

*Bordetella* infection can be efficiently prevented through vaccination. The current study investigated the effects of an extract of *Cochinchina momordica* seed (ECMS) combined with oil on the immune responses to the inactivated *Bordetella* vaccine in mice. Serum IgG and IgG1 level was significantly increased in ECMS-oil group compared to any other group (*P* < 0.05) 2 weeks after immunization, while groups ECMS200 *μ*g/400 *μ*g-oil had a markedly higher level of serum IgG2b and IgG3 than any other groups (*P* < 0.05). Moreover, lipopolysaccharide/ConA-stimulated proliferation of splenocytes was significantly enhanced in ECMS 400 *μ*g-oil immunized mice in comparison with mice in any other group (*P* < 0.05). RT-PCR assay revealed that while ECMS800 *μ*g-oil group had significantly higher levels of serum IL-4, IL-10, Toll-like receptor (TLR)2, and IL-1 beta than any other group (*P* < 0.05), the levels of serum IL-2, IL-4, and IL-10 were markedly increased in ECMS 400 *μ*g-oil group as compared to any other groups (*P* < 0.05). Blood analysis showed that ECMS800 *μ*g-oil and oil groups had a significantly higher number of immunocytes than any other groups (*P* < 0.05). There were significant differences in the number of IgG+, IgG2b+, and IgA+ cells in the lung between ECMS800 *μ*g-oil group and any other groups (*P* < 0.05). Western blot analysis demonstrated that stimulation with ECMS 25 *μ*g/mL or 50 ng/mL led to a significant increase in the expression of TLR2, MyD88, and NF-*κ*B in Raw264.7 cells (*P* < 0.05). Compared with any other group, the expression of MyD88 was markedly increased in the cells stimulated with ECMS 50 ng/mL, as indicated by the RT-PCR analysis (*P* < 0.05). Overall, we observed that ECMS-oil efficiently enhanced the humoral or cellular immune responses against *Bordetella* and suggested that the mechanism of adjuvant activity of ECMS-oil might involve TLR2/MyD88/NF-*κ*B signaling pathway.

## 1. Introduction

*Bordetella bronchiseptica* is an etiological agent of bronchial *Bordetella* disease in the respiratory tract of mammals. The infection can occur in every season but is more prevalent in spring and fall. Transmission occurs via the nasal secretion of infected animals such as rabbits and pigs. In rabbits, infection with *B. bronchiseptica* can lead to acute death around the weaning time, as well as bronchitis, rhinitis, and pustular pneumonia in adulthood. Bronchial *Bordetella* disease can spread quickly, and it is very hard to control and eliminate, thereby causing substantial economic losses [1, 2]. Vaccination is considered the most efficient method for preventing *Bordetella* infection. At present, studies on vaccine adjuvants for bronchial *Bordetella* disease are just beginning. Although an attenuated vaccine for *B. bronchiseptica* is capable of eliciting an immune response, its application is limited due to issues of biosafety, transportation, and storage [3]. While the inactivated *Bordetella* vaccine has been proved to be safe, the weak immunogenicity of *Bordetella* antigens needs to be overcome by using adjuvants for veterinary *B. bronchiseptica* vaccine [4]. Currently, adjuvants still have some shortcomings, such as weak immune responses and short duration of antibody response [1].

Spreng is found mainly in the wild and grown in several provinces, such as Hubei, Guangxi, and Sichuan. And its mature seed is *Cochinchina momordica*. The herbal medicine *cochinchinensis* could be prepared from spreng by removing its pulp and drying the seeds. It has been reported that compared with QuilA, the extract of *C. momordica* seed (ECMS) is a more suitable adjuvant for vaccines because of its lower hemolytic activity. A previous study has shown that an ECMS oil emulsion can greatly facilitate the immune effects of various pathogens in animals [5]. It has also been demonstrated that an appropriate dose of ECMS can be used as an adjuvant with OVA antigen to boost humoral and cellular immunity in mice [6]. We previously showed that ECMs-oil adjuvant could improve the immune effect of *Bordetella* vaccine in rabbits. In this study, we investigated the mechanism of adjuvant activity of ECMS-oil in mice. Moreover, we performed stimulation experiments using Raw264.7 macrophages to identify the pathway underlying the adjuvant activity of ECMS-oil.

## 2. Materials and Methods

### 2.1. Adjuvants and Antigens

The ECMS and oil (mineral oil) were obtained from the Laboratory of Veterinary Medicine at Zhejiang University and Connaught Times Wei Biotechnology Co., Ltd., respectively. The endotoxin level of ECMS was analyzed by using the Limulus amebocyte lysate assay and was found to be <0.5 unit/mL [6]. FX strain of *B. bronchiseptica* [1] was cultured on sheep blood agar and tryptone soya broth with 5% bovine calf serum at 37°C in a rotary shaker at 200 rpm. To prepare the vaccine antigen, *B. bronchiseptica* was quantified using the plate counting method, cultured by GMP-certified fermentation, and annihilated with 0.2% formalin for 36 hr [1]. After that, the antigen was stored at 4°C. The whole cell protein was prepared for enzyme-linked immunosorbent assay (ELISA). Murine macrophages RAW264.7 were purchased from the Cell Bank of the Chinese Academy of Sciences in Shanghai.

### 2.2. Experimental Animals and Ethics Statement

Balb/c female mice (15–20 g) were purchased from the Zhejiang Academy of Agricultural Sciences. All mice were subjected to 1 week of acclimatization prior to experiments. Ethical approval was obtained from the ethics committee of the Zhejiang Academy of Agricultural Sciences (ethics protocol no. 2021ZAASLA11), and all animal experiments were carried out according to the guidelines issued by Zhejiang Farm Animal Welfare Council.

### 2.3. Vaccine Preparation

The vaccine components are listed in Table 1. Briefly, the oil-mixed adjuvants were emulsified twice for immunization at 60 Hz for 90 min.

### 2.4. Animal Immunization

A total of 144 female balb/c mice were categorized into six groups, with each 24 mice. Mice were immunized subcutaneously with 0.2 mL (1.6 × 10^7^ CFU) of *B. bronchiseptica* alone (group D), *B. bronchiseptica* in phosphate buffer saline (PBS) with ECMS (group A1, 200 *μ*g + oil; group A2, 400 *μ*g + oil; group A3, 800 *μ*g + oil), oil (group B), or 800 *μ*g of ECMS (group C) on day 1 and 15 (Table 1). Two weeks after the second immunization, sera, and splenocytes were collected for antibody measurement and proliferation estimation, respectively. All mice were killed by cervical dislocation at the end of the experiment.

### 2.5. Quantitative Measurement of IgG and Its Subclasses

IgG and its subclasses were measured using the ELISA kit (eBioscience, ThermoFisher Scientific) (IgG, Cat No. 39-50400-65; IgG1, Cat No. 39-50410-65; IgG2b, Cat No. 39-50430-65; IgG3, Cat No. 39-50440-65). Briefly, the plates were coated with capture antibody diluted in ELISA coating buffer (100 *μ*L/well) for 12–16 hr at 4°C. Then, plates were washed and blocked in blocking buffer (250 *μ*L/well) at RT for 2 hr. The standards were two-fold serial diluted with 1× Assay Buffer A to generate a standard curve. One hundred microliters of 1× Assay Buffer A was applied to each standard well, and the reconstituted standard (100 *μ*L in duplicate) was added to well A1 and A2. Thereafter, the contents in the wells were subjected to repeated aspiration and ejection, and 100 *μ*L mixture was transferred from wells A1 and A2 to wells B1 and B2, respectively. The concentration of standard 1 was as follows: S1 = 100 ng/mL (IgG), S1 = 200 ng/mL (IgG1), S1 = 250 ng/mL (IgG2b), and S1 = 125 ng/mL (IgG3). The previous procedure was repeated five times. The blank wells and sample wells were filled with 100 *μ*L/well of 1× Assay Buffer A and 90 *μ*L/well of 1× Assay Buffer A, respectively. Prediluted serums (10 *μ*L/well) and diluted detection antibodies (50 *μ*L/well) were sequentially applied into the appropriate wells. After incubation for 2 hr at RT, plates were washed four times. Finally, the substrate (100 *μ*L/well) was added to the plates and incubated for 15 min at RT. The stop solution (100 *μ*L/well) was then added to terminate the reaction. Optical densities at 450 nm were measured on an ELISA reader.

### 2.6. Splenocyte Proliferation Assay

After the second immunization, half of the mice from each group were randomly selected and sacrificed for yielding spleens. The spleens in Roswell Park Memorial Institute (RPMI) 1640 were finely ground individually into homogeneous cell suspensions and then centrifuged at 380 × *g* for 10 min at 4°C. After washing, the cell pellet was resuspended into RPMI 1640 containing 10% fetal bovine serum (FBS), 100 *μ*g/mL penicillin, and 100 *μ*g/mL streptomycin. The cell viability exceeding 95% was estimated using trypan blue exclusion test on a hemocytometer. The proliferation of splenocytes was assessed as previously reported [6]. In short, the cells were seeded into a 96-well plate at a density of 5 × 10^5^ cells per well and incubated with ConA (10 *μ*g/mL) (Sigma, St. Louis, USA) or lipopolysaccharide (LPS) (5 *μ*g/mL) for 68 hr at 37°C under 5% CO_2_. 3-(4,5-dimethylthiazol-2-yl)-2,5-diphenyltetrazolium bromide assay was performed to detect T and B-cell proliferation. In the assay, the precipitates were solubilized by incubation with 144 *μ*L dimethyl sulfoxide containing 6 *μ*L of 1N HCl for 15 min at 37°C. The absorbance at 490 nm was estimated by ELISA. Stimulation index was calculated as the ratio between the absorbance of mitogen-stimulated cultures and the absorbance of nonstimulated cultures.

### 2.7. Measurement of Serum and Intracellular Cytokines

Blood samples were collected 2 weeks post the second immunization, and the levels of serum IL-2, IL-4, and IL-10 were determined by an ELISA kit (RD SystemsS Co., Ltd.). The levels of TNF-*α* and IL-12 in the macrophages were measured using an ELISA kit (Multi Sciences, Ltd.). Fifty microliters of the assay diluent and 50 *µ*L of the standards or samples were sequentially added to each well and incubated for 2 hr at 37°C. Thereafter, the plates were washed and then coated with 100 *μ*L of each mouse cytokine (IL-2, IL-4, IL-10, TNF-*α*, and IL-12) at 37°C for 2 hr. After washing, the reaction was estimated as described earlier. Finally, the cytokine levels in the samples were extrapolated from the standard curve.

### 2.8. Blood Analysis

Half of the mice from each group were randomly selected for blood analysis. The whole blood in anticoagulant was collected from eyes 2 weeks after the second immunization. White blood cells (WBC), lymphocytes (squamous cell carcinoma (SCC)), intermediate cells (Merkel cell carcinoma (MCC) including monocytes, eosinophils, and basophils), and neutrophil granulocytes (large-cell carcinoma (LCC)) were counted with an automated hematology analyzer (Sysmex, pocH-100iV, JP). The results were presented as ×10^3^/*μ*L.

### 2.9. Real-Time PCR

Total RNA was extracted from the blood samples, including WBC, lymphocytes (SCC), intermediate cells (MCC including monocytes, eosinophils, and basophils), and neutrophil granulocytes (LCC) or Raw264.7 cells using PureLink RNA Mini kit and quantitatively measured by optical density determination at 260 nm. One microgram of RNA from each sample was reverse-transcribed into cDNA with the Promega cDNA synthesis kit following the manufacturer's protocol.

Real-time PCR was conducted to quantify the mRNA expression of targets, such as IL-4, IL-10, Toll-like receptor (TLR)2, TLR4, IL-1 beta, ASC-1, NF-kB, and MyD88. PCR primers used in this study are presented in Table 2. GAPDH was included as an internal reference. 2^−⊿⊿CT^ method was used to determine the relative expression level [7], and each assay was conducted at least in triplicate.

### 2.10. Immunohistochemistry

IgG+, IgG2b+, and IgA+ cells in the lung were detected, as described by Xiao et al. [1]. The immunized mice were sacrificed for yielding lungs 2 weeks post the second immunization. The lungs were fixed immediately with 10% neutral-buffered formalin (pH = 7.4), dehydrated in gradients of alcohol (80%–100%), followed by xylene and immersed into paraffin. Paraffin-embedded tissues were sliced into 4 *μ*m sections, deparaffinized with xylene, and subjected to hydration through decreasing concentrations of ethanol (100%–70%). Then, the slices were washed and preincubated with 3% hydrogen peroxide for 10 min. After being rinsed three times with PBS, slices were subjected to incubation with anti-IgG antibody (Abcam), anti-IgG2b antibody (Abcam), or anti-IgA antibody (DAKO) in a moist chamber at 37°C for 1 hr, followed by incubation with horseradish peroxidase-conjugated goat antirabbit IgG at 37°C for 30 min. Thereafter, the slices were stained with 3,3-diaminobenzidine chromagen, and the stained slices were examined by light microscopy. Finally, slices were subjected to counterstaining using hematoxylin. As a negative control, two randomly selected slices were incubated with PBS rather than primary antibodies.

### 2.11. Cell Proliferation Assay

In order to further verify the adjuvant mechanism of ECMS, mouse macrophages were used to determine the relevant experimental indexes and pathway types. The macrophages were grown in Dulbecco's modified Eagle's medium supplemented with 10% FBS and 100 *μ*g/mL of penicillin and streptomycin. Cells were plated on a 6-well plate (5 × 10^6^ cells/well) and cultivated for 24 hr at 37°C under 5% CO_2_. Afterward, the complete medium with ECMS or LPS at a predetermined concentration was applied into each well. A final concentration of 25 *μ*g/mL or 50 ng/mL for ECMS and the final concentration of 10 *μ*g/mL for LPS were used for this assay. LPS and medium alone were chosen as the positive control and negative control (CK), respectively.

### 2.12. Western Blotting

Total protein was extracted from frozen cells with radio-immunoprecipitation assay buffer. Extracted protein was quantified using a BCA assay kit. Protein samples were resolved by SDS–PAGE and then electro-blotted onto polyvinylidene fluoride membranes. The membrane was blocked and then incubated overnight at 4°C with the following primary antibodies: anti-TLR2 (1 : 1,000, ABCAM), anti-MyD88 (1 : 1,000, ABCAM), anti-NF-*κ*B (1 : 1,000, ABCAM), or anti-Actin (1 : 10,000, Proteintech). On the next day, the membrane was subjected to incubation with goat antimouse IgG (1 : 10,000, ABBKINE) for 1 hr at RT. Immunoreactive proteins were visualized using enhanced chemiluminescence and analyzed with an image analyzer.

### 2.13. Challenge Experiments

Twelve mice in each group were randomly selected for the challenge experiment. Two hundred microliters of *B. bronchiseptica* (1.6 × 10^6^ CFU) were used for the challenge through intraperitoneal injection in each mouse. All the mice were observed for survival during 2 weeks after the attack and the death were recorded.

### 2.14. Statistics

All data were expressed as mean ± SD. Statistical analysis was conducted using one-way (analysis of variance) followed by Tukey's honestly significant difference test. A *P* value of <0.05 was statistically significant.

## 3. Results

### 3.1. The Levels of Serum IgG and Its Subclasses

**Figure 1:**
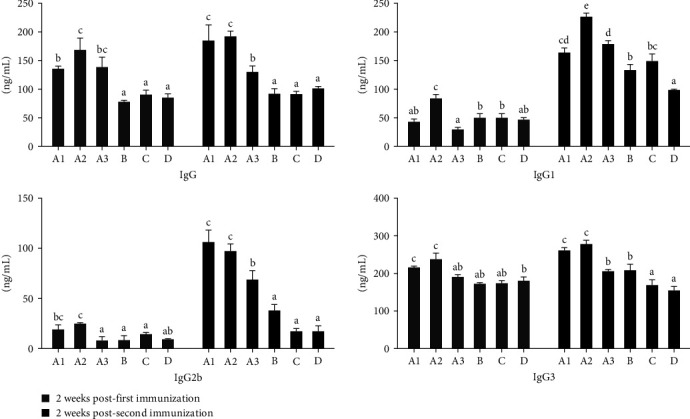
The levels of serum IgG and its subclasses. The horizontal and vertical coordinates of the graph stand for the level of antibody and different groups. Different letters above the bar indicate the presence of significant differences among the groups (*P* < 0.05). Groups A1, A2, and A3 stand for (ECMS200 *μ*g-oil, ECMS 400 *μ*g-oil, and ECMS800 *μ*g-oil) group. Group B stands for oil group, group C stands for EMCS800 *μ*g group, group D stands for phosphate buffer saline group. Serum IgG level during 2 weeks post the first immunization was significantly increased in ECMS-oil groups (A1, A2, A3) compared with oil group (B), ECMS group (C), or control group (D) (*P* < 0.05). Meanwhile, ECMS 400 *μ*g-oil group (A2) had a significantly higher level of serum IgG1 than any other groups during 2 weeks after the first and second immunization (*P* < 0.05). Moreover, groups A1, A2, and A3 had a markedly higher level of serum IgG2b than any other groups (B, C, D) during 2 weeks post the second immunization (*P* < 0.05), while serum IgG3 level during 2 weeks post the first and second immunization was substantially elevated in groups A1 and A2 as compared to any other groups (A3, B, C, and D) (*P* < 0.05).

Mice were immunized subcutaneously twice to examine the humoral immune responses induced by ECMS oil. Two weeks after the first and second immunization, the concentrations of serum IgG and its subclasses were monitored by ELISA. As depicted in Figure 1, serum IgG level during 2 weeks post the first immunization was significantly increased in ECMS-oil groups (A1, A2, A3) compared with oil group (B), ECMS group (C), or control group (D) (*P* < 0.05). Meanwhile, ECMS 400 *μ*g-oil group (A2) had a significantly higher level of serum IgG1 than any other groups during 2 weeks after the first and second immunization (*P* < 0.05). Moreover, groups A1, A2, and A3 had a markedly higher level of serum IgG2b than any other groups (B, C, D) during 2 weeks post the second immunization (*P* < 0.05), while serum IgG3 level during 2 weeks post the first and second immunization was substantially elevated in groups A1 and A2 as compared to any other groups (A3, B, C, and D) (*P* < 0.05).

### 3.2. Splenocyte Proliferation Analysis

**Figure 2:**
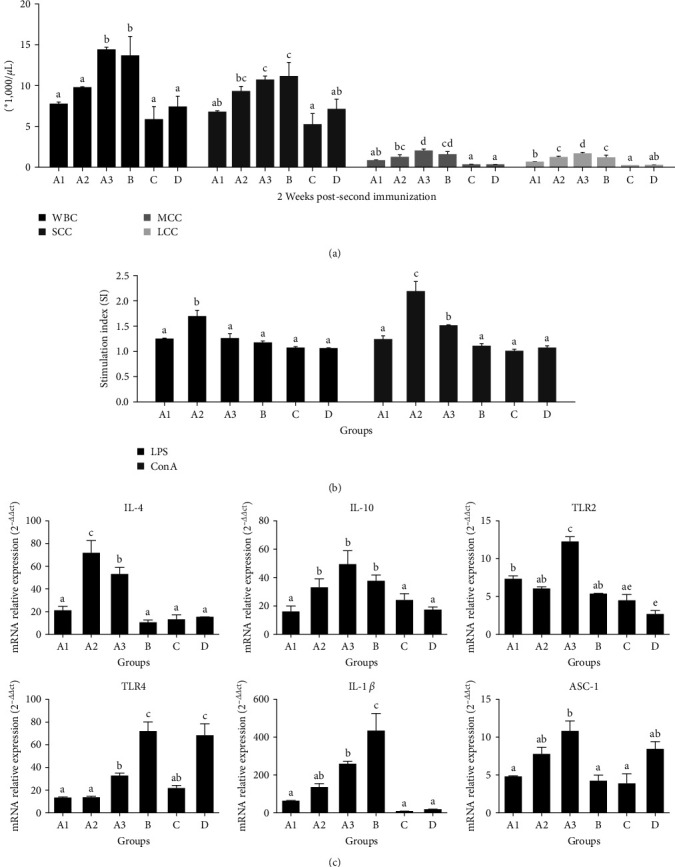
Results of blood analysis, splenocyte proliferation analysis, and the expression level of cytokines and the relevant factors. Different letters above the bar indicate the presence of significant differences among the groups (*P* < 0.05). Groups A1, A2, and A3 stand for (ECMS200 *μ*g-oil, ECMS 400 *μ*g-oil, and ECMS800 *μ*g-oil) group. Group B stands for oil group, group C stands for EMCS800 *μ*g group, group D stands for phosphate buffer saline group.

As illustrated in Figure 2(b), LPS-stimulated proliferation of splenocytes was markedly promoted in group A2 (ECMS 400 *μ*g-oil) as compared to any other groups (*P* < 0.05). Likewise, ConA-stimulated proliferation of splenocytes in groups A2 and A3 was significantly stronger than that in any other groups (*P* < 0.05).

### 3.3. The Serum Levels of Cytokines

The levels of serum IL-2 and IL-10 were significantly increased in group A2 as compared to groups B, C, and D (*P* < 0.05). Likewise, group A2 had a significantly higher level of IL-4 than any other group except group C (*P* < 0.05) (see *Supplementary 1*).

### 3.4. Blood Analysis

As shown in Figure 2(a), the number of WBCs in groups A3 and B were markedly greater than that in the other groups (*P* < 0.05). Likewise, the number of leukomonocyte (SCCs) was significantly elevated in groups A3 and B as compared to all other groups (excluding A2) (*P* < 0.05). Moreover, we observed that while the number of MCCs in group A3 was obviously higher than that in groups A1, A2, C, and D (*P* < 0.05), the number of LCCs was significantly increased in group A3 compared with all other groups, including the control (*P* < 0.05).

### 3.5. The Expression Level of Cytokines and the Relevant Factors

We examined the expression of cytokines and the other relevant factors in blood samples, including IL-4, IL-10, TLR2, TLR4, IL-1 beta, and ASC-1. As depicted in Figure 2(c), IL-4 level in groups A2 and A3 was markedly higher than that in any other groups (*P* < 0.05), and IL-10 level was significantly upregulated in groups A2, A3, and B as compared to any other groups (*P* < 0.05). Likewise, TLR2 level in group A3 was markedly higher than that in any other group (*P* < 0.05), and TLR4 level was significantly increased in groups B and D compared with the other groups (*P* < 0.05). Moreover, the level of IL-1 beta in group B was markedly higher than that in all other groups (*P* < 0.05), and ASC-1 level was significantly upregulated in group A3 as compared to all other groups (excluding groups A2 and D (*P* < 0.05).

### 3.6. IgG+, IgA+, and IgG2b+ Cells in the Lung

**Figure 3:**
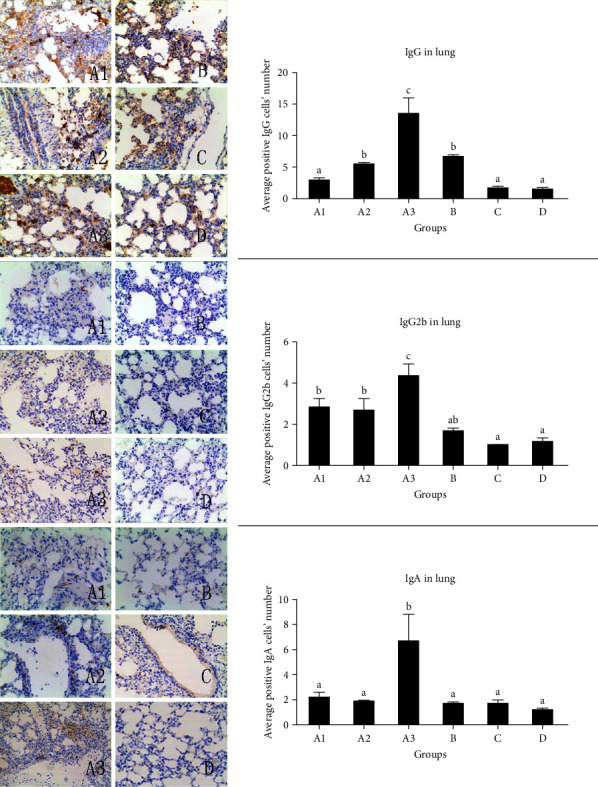
Results of IgG+, IgA+, and IgG2b+ cells in the lung. Different letters above the bar indicate the presence of significant differences among the groups (*P* < 0.05). Groups A1, A2, and A3 stand for (ECMS200 *μ*g-oil, ECMS 400 *μ*g-oil, and ECMS800 *μ*g-oil) group. Group B stands for oil group, group C stands for EMCS800 *μ*g group, group D stands for phosphate buffer saline group.

As illustrated in Figure 3, there were significant differences in the number of IgG+ cells in the lung between group A3 and the other groups (*P* < 0.05). Similarly, there were significant differences in the number of IgA+ cells between group A3 and all other groups (*P* < 0.05), and no significant difference in the number of IgG2b+ cells was found between group A3 and all other groups.

### 3.7. Challenge Experiments

In challenge experiments using *B. bronchiseptica*, we observed that among all the groups, groups A2 and A3 displayed the highest survival rate (75%), while groups A1 and B had a less survival rate (50%). And the survival rate in groups C and D was found to be 33.3% and 8.33%, respectively (see *Supplementary 2*).

#### 3.7.1. The Expression of TNF-*α*, IL-12, TLR2, MyD88, and NF-*κ*B in Raw264.7 Cells

**Figure 4:**
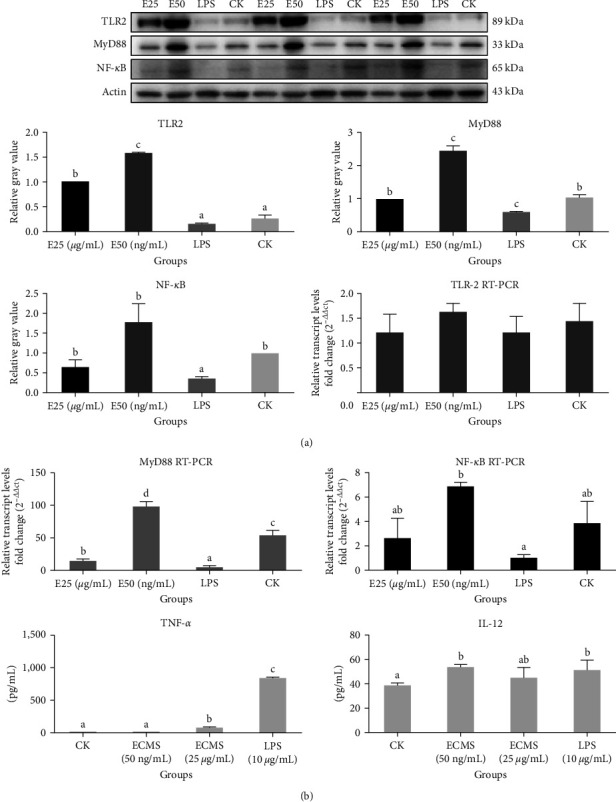
The results of western blot TLR2, MyD88, NF-*κ*B, and TNF-*α*, IL-12 concentration from Raw264.7 cell supernatant. Different letters above the bar indicate the presence of significant differences among the groups (*P* < 0.05). The horizontal and vertical coordinates of the graph stand for the level of cytokines and different groups in (a). The horizontal and vertical coordinates of the graph stand for the relative transcript levels of cytokines and different groups in (b) or level of cytokines and different groups in (b). Western blot analysis revealed that induction with 50 ng/mL or 25 *μ*g/mL ECMS led to an increased expression of TLR2, while the expression of MyD88 and NF-*κ*B was increased in the macrophages induced with 50 ng/mL ECMS (a). RT-PCR assay showed that while MyD88 expression was increased in ECMS-induced macrophages (50 ng/mL) as compared to cells in any other groups (*P* < 0.05), the expression level of NF-*κ*B in ECMS-induced macrophages (50 ng/mL) was higher than that in lipopolysaccharide group (*P* < 0.05) (b).

Western blot analysis revealed that induction with 50 ng/mL or 25 *μ*g/mL ECMS led to an increased expression of TLR2 in Raw264.7 cells, while the expression of MyD88 and NF-*κ*B was increased in the macrophages induced with 50 ng/mL ECMS (Figure 4(a)). Notably, there were significant differences in the concentrations of TNF-*α* and IL-12 between ECMS-stimulated cells (25 *μ*g/mL or 50 ng/mL) and the controls (*P* < 0.05) (Figure 4(b)). Moreover, RT-PCR assay showed that while MyD88 expression was increased in ECMS-induced macrophages (50 ng/mL) as compared to cells in any other groups (*P* < 0.05), the expression level of NF-*κ*B in ECMS-induced macrophages (50 ng/mL) was higher than that in LPS group (*P* < 0.05) (Figure 4(b)).

## 4. Discussion

Unlike the traditional vaccines, new generation of vaccines comprising recombinant proteins and DNA in conjunction with improved adjuvants do not trigger an immunogenic response [8]. Experimental tests have shown that several adjuvants can initiate antibody responses and cause adverse reactions, indicating that their vaccine applicability is limited [9, 10]. It has been reported that *B. bronchiseptica* may cause respiratory problems in animals, including kennel cough in dogs, snuffles in rabbits, atrophic rhinitis, and bronchopneumonia in swine [11]. It has also been demonstrated that serum agglutination titer is associated with protection against *B. parapertussis* [12]. The subtypes of IgG immunoglobulins IgG1, IgG2b, and IgG3 provide immunity against most infectious agents [13]. In this study, we found that the levels of total IgG, IgG1, IgG2b, and IgG3 in ECMS 400 *μ*g-oil group were the highest among all the groups, showing that ECMS 400 *μ*g-oil could induce humoral immune response in mice. As an adjuvant, Quil A extracted from the bark of *Quillaja saponaria* Molina had synergistic effect in an oil emulsion and promoted the production of IgG1, IgG2a, IgG2b, and IgG3 in OVA-induced mice [13]. An elevated Th1 reaction in mice acts as a prerequisite for production of cytotoxic T-lymphocytes and leads to an increase in the expression of IL-2 as well as IgG2a, IgG2b, and IgG3. Th2 response can be distinguished from Th1 response based on IL-4, IL-10, and augmented IgG1. Herein, we showed that ECMS 400 *μ*g-oil significantly upregulated the expression of IL-2, IL-4, and IL-10, indicating that it could enhance both Th1 and Th2 responses in the immunization. This finding was consistent with the previous observation that adjuvants significantly influenced the immune responses through triggering Th1 or Th2 response [14]. The present study demonstrated that ECMS 400 *μ*g-oil remarkably promoted ConA/LPS-stimulated proliferation of splenocytes in the immunized mice, suggesting that it may notably enhance the activities of T and B cells [15].

T and B cells are responsible for the adaptive immune recognition [16]. In this study, we observed that ECMS800 *μ*g-oil (A2) and oil groups (B) had a significantly higher number of WBCs than any other groups. Lymphocytes mature upon antigen stimulation and subsequently induce cellular immune responses against the antigens. Notably, we found that while the number of SCCs was significantly increased in ECMS800 *μ*g-oil (A3) and oil (B) groups as compared to any other groups (excluding ECMS 400 *μ*g-oil group), ECMS800 *μ*g-oil (A3) and oil (B) groups had more intermediate cells than any other groups. Intermediate cells could be a class of rarer cells comprising eosinophils, monocytes, basophils, blasts, and other white cell precursors. The whole immune level has been improved 2 weeks after immunization indicating the whole immune cell level could have been improved. The present study showed that the number of intermediate cells (MCC) was obviously elevated in ECMS800 *μ*g-oil (A3) and oil (B) groups compared with any other groups. More interestingly, we found that ECMS800 *μ*g-oil group had a markedly higher number of neutrophil granulocytes (LCC) than any other group. It has been shown that the novel Matrix-M™ adjuvant can potentiate immune responses against an influenza vaccine. Neutrophils stimulate quick and potent immune responses, thus facilitating phagocytosis, chemotaxis, and bactericidal activities. The previous finding may explain mechanism of adjuvant activity of ECMS-oil. Further research should be focused on identification of the triggering factor.

To elucidate the underlying mechanism, we analyzed the mRNA expression of serum IL-4, IL-10, TLR2, TLR4, IL-1 beta, and ASC-1 2 weeks post the second immunization. The analysis revealed that ECMS 400 *μ*g-oil efficiently enhanced the expression of IL-4, while ECMS 400 *μ*g/800 *μ*g-oil significantly promoted the expression of IL-10 and ASC-1 TLRs act as pattern recognition receptors (PRRs) for recognizing the conserved pathogen-associated molecular patterns [17, 18]. TLRs constitute the first-line defense against pathogenic infections and play a crucial role in the innate immune response. The cascade of multiple signaling events can induce pro-inflammatory cytokines, eliciting an efficient adaptive immunity [19, 20]. TLR-MyD88 signaling has been shown to efficiently regulate TLR-mediated immune response. Herein, we observed that ECMS800 *μ*g-oil efficiently enhanced the expression of TLR2, suggesting that the mechanism underlying the activity of ECMS-oil may involve TLR2/MyD88/NF-*κ*B signaling pathway. Furthermore, we analyzed the expression of the cytokines in Raw264.7 cells and obtained data supporting that TLR2/MyD88/NF-*κ*B pathway may underlie the mechanism of ECMS-oil.

The secretory immunoglobulins IgA and IgG present in mucosal surface are responsible for preventing the invasion of microorganisms such as viruses and bacteria. Respiratory tract and alimentary tract mainly contain secretory IgA that is critically involved in mucosal humoral immune response [21]. It has been reported that IgA acts as an important factor in the control of pertussis infection [22]. The present study demonstrated that there were significant differences in the number of IgA+ cells between group ECMS800 *μ*g-oil and the other groups. Recently, mucosal IgG has been found to function in host defense [23]. Notably, the current study identified significant differences in the number of IgG-positive cells between ECMS 400/800 *μ*g-oil and oil groups and the other groups. IgG subclasses differ in the abilities to enhance phagocytosis, with a relative efficacy descending from IgG2b to IgG2a and IgG1 [24]. Moreover, we found that IgG2b positive cells in the lungs in groups ECMS200/400/800 *μ*g-oil were significantly different from those in all other groups (excluding oil group).

The extracellular matrix (ECM) components function like danger-associated molecular patterns (DAMPs) by directly interacting with PRRs such as TLRs and inflammasomes [25]. Upon interactions between ECM and PRRs, DAMPs derived from ECM autonomously elicit sterile inflammation or prolong the responses induced by pathogens via generation of proinflammatory mediators as well as recruitment of leukocytes to sites of infection and damage. Mechanism of adjuvant activity of ECMS-oil might involve TLR2/MyD88/NF-*κ*B-dependent stimulation of macrophages. Activated PRRs can elicit an inflammatory cascade involving the expression and secretion of cytokines, the recruitment of leukocytes to sites of infection and injury, as well as switch to adaptive immune response for pathogen clearance in the case of tissue injury [26].

Furthermore, we found that groups oil +ECMS 400 *μ*g/800 *μ*g had the highest survival rate (75%) among all the groups, while oil +ECMS 200 *μ*g and oil groups displayed a less survival rate (50%). And IgG1 is supposed to play an important role in antibacterial activities [27]. Taken together, these results suggested that ECMS 400 *μ*g/800 *μ*g-oil could provide a first line of defense against *Bordetella bronchiseptica*, while potentially serving as a safe and efficient commercial vaccine adjuvant.

## 5. Conclusion

The present study presented data showing an interdependent influence of ECMS and oil on humoral and cellular immune responses. Hence, ECMS-oil could become an alternative for improving vaccination against *B. bronchiseptica* in mice. Further analysis indicated that mechanism of adjuvant activity of ECMS-oil might involve TLR2/MyD88/NF-*κ*B signaling pathway. Overall, this study suggested that ECMS 400 *μ*g/800 *μ*g-oil could provide a first line of defense against *B. bronchiseptica*, while it may potentially be used as a safe and efficient commercial vaccine adjuvant.

## Figures and Tables

**Table 1:** Adjuvant vaccines.

Groups	Preparation dose	
ECMS + oil (A1, A2, A3)	150 *μ*L oil + 2 *μ*L Tween+ECMS (200, 400, 800 *μ*g)	1.6 × 10^7^ CFU in PBS 50 *μ*L
Oil (B)	150 *μ*L oil + 2 *μ*L Tween	1.6 × 10^7^ CFU in PBS 50 *μ*L
ECMS (C)	ECMS(800 *μ*g) in PBS 100 *μ*L	1.6 × 10^7^ CFU in PBS 100 *μ*L
PBS (D)		1.6 × 10^7^ CFU in PBS 200 *μ*L

PBS: phosphate buffer saline.

**Table 2:** Real-time PCR primers.

No.	Gene	Sequence	Product length (bp)
1	GAPDH-F	CCCACAGTAAATTCAACGGCAC	87
	GAPDH-R	CATTGGGGTTAGGAACACGGA	
2	IL4-F	CTAGTTGTCATCCTGCTCT	102
	IL4-R	AATAAAATATGCGAAGCACCT	
3	IL10-F	CTCCCCTGTGAAAATAAGAGC	105
	IL10-R	GACTCAATACACACTGCAGGT	
4	TLR2-F	CATGAAAAGCCTTGACCTGT	92
	TLR2-R	AGAATAAAAGGCGTCTCCCTC	
5	TLR4-F	CCATCATTATGAGTGCCAA	85
	TLR4-R	AAGCCAAGAAATATACCATCG	
6	IL-1 beta-F	ATTAGACAACTGCACTACAGG	103
	IL-1 beta-R	ATACTTTAGGAAGACACGGAT	
7	ASC-1-F	GAAGCTGCTGACAGTGCAAC	112
	ASC-1-R	AGGAGGAACAGTTAAGCGCC	
8	NF-kb-F	CTTCGATGGCACTGCTGTCA	81
	NF-kb-R	TGGAGTTCCTGGTAGAACGC	
9	MyD88-F	AGAGCTGCTGGCCTTGTTAG	96
	MyD88-R	AAGCACGTTTCCTCACCGAT	

## Data Availability

The data used to support the findings of this study are included within the article and the supplementary information files.

## Supplementary Materials

Supplementary 1 Cytokine concentration of IL-2, IL-4 and IL-10.

Supplementary 2 Survival protection after Bb challenge.
