# Supplementary material for: Adjuvant Efficacy of the ECMS-Oil on Immune Responses against Bordetella bronchiseptica in Mice through the TLR2/MyD88/NF-κB Pathway
Source: J Immunol Res. 2023 Mar 27;2023:1011659. doi: 10.1155/2023/1011659 (PMC10234371; doi:10.1155/2023/1011659)
Supplement: Supplementary file 1 [file 1011659.f1.docx]

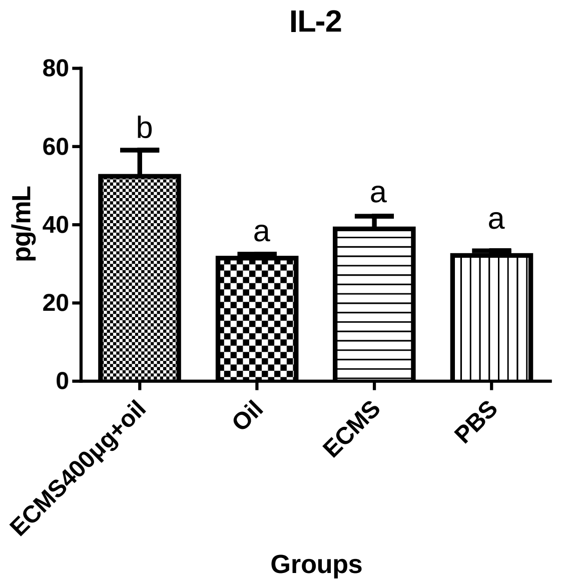
IL-2
pg/mL
0
20
40
60
80
b
a
a
a
ECMS400μg+oil
Oil
ECMS
PBS
Groups

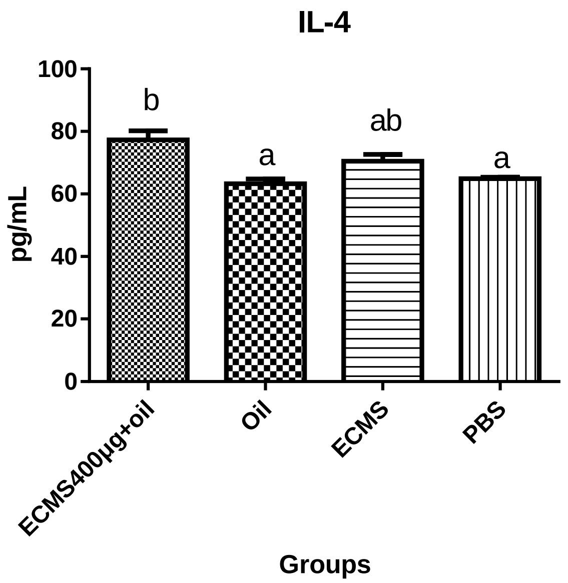
IL-4
pg/mL
0
20
40
60
80
100
b
a
ab
a
ECMS400μg+oil
Oil
ECMS
PBS
Groups

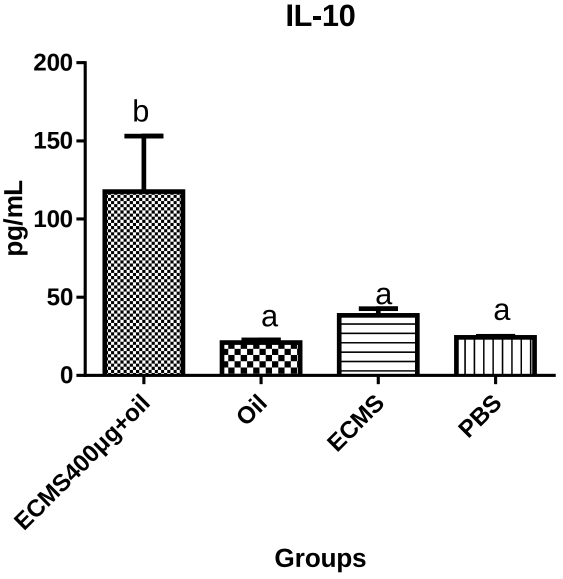
IL-10
pg/mL
0
50
100
150
200
b
a
a
a
ECMS400μg+oil
Oil
ECMS
PBS
Groups
