# Supplementary material for: Adjuvant Efficacy of the ECMS-Oil on Immune Responses against Bordetella bronchiseptica in Mice through the TLR2/MyD88/NF-κB Pathway
Source: J Immunol Res. 2023 Mar 27;2023:1011659. doi: 10.1155/2023/1011659 (PMC10234371; doi:10.1155/2023/1011659)
Supplement: Supplementary file 2 [file 1011659.f2.docx]

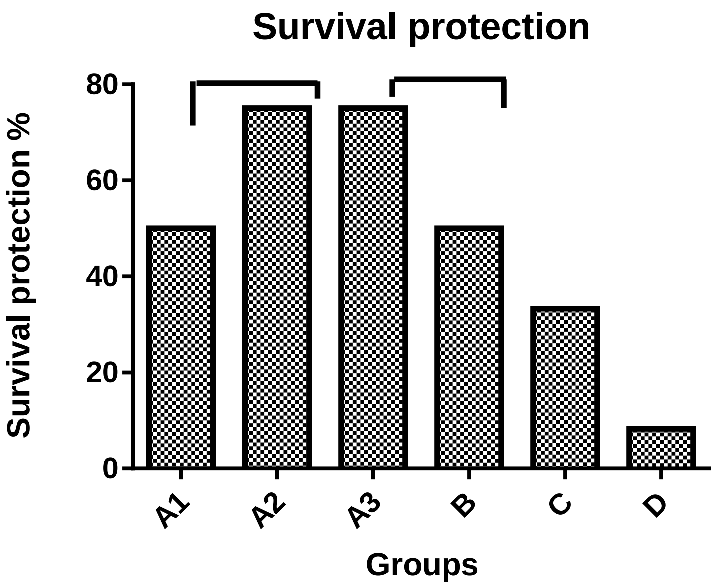

Survival protection
Survival protection %
80
60
40
20
0
A1
A2
A3
B
C
D
Groups
